# Influenza B infection and Kawasaki disease in an adolescent during the COVID-19 pandemic: a case report

**DOI:** 10.5935/0103-507X.20210041

**Published:** 2021

**Authors:** Jéssica de Oliveira Silveira, Mariana Grando Pegoraro, Juliana Ferreira Ferranti, Albert Bousso, Tadeu Silveira Martins Renattini

**Affiliations:** 1 Pediatric Intensive Care Unit, Hospital Israelita Albert Einstein - São Paulo (SP), Brazil.; 2 Pediatric Intensive Care Unit, Hospital Municipal Vila Santa Catarina Dr. Gilson de Cássia Marques de Carvalho - São Paulo (SP), Brazil.; 3 Pediatric Intensive Care Unit, Instituto da Criança, Faculdade de Medicina, Universidade de São Paulo - São Paulo (SP), Brazil.

**Keywords:** Mucocutaneous lymph node syndrome, Influenza B virus, Coronavirus infections, COVID-19, SARS-CoV-2, Respiratory insufficiency, Adolescent, Síndrome de linfonodos mucocutâneos, Vírus da Influenza B, Infecções por coronavírus, COVID-19, SARS-CoV-2, Insuficiência respiratória, Adolescente

## Abstract

We report a case of Influenza B infection and Kawasaki disease in an adolescent, diagnosed during the COVID-19 pandemic. An asthmatic female adolescent presented with fever and flu-like symptoms for 7 days and was admitted with acute respiratory failure requiring mechanical ventilation. She progressed with hemodynamic instability responsive to vasoactive drugs. Antibiotic therapy and support measures were introduced, showing progressive hemodynamics and respiratory improvement, however with persistent fever and increased inflammatory markers. During the hospitalization, she developed bilateral non-purulent conjunctivitis, hand and feet desquamation, strawberry tongue, and cervical adenopathy, and was diagnosed with Kawasaki disease. She was prescribed intravenous immunoglobulin and, due to the refractory clinical conditions, corticosteroid therapy was added; 24 hours later, the patient was afebrile. No coronary changes were found. A full viral panel including COVID-19 C-reactive protein and serology could only isolate the Influenza B virus. During the hospitalization, she was diagnosed with pulmonary thromboembolism; coagulopathies were investigated, and she was diagnosed with heterozygous factor V Leiden mutation. There is a potential association between Kawasaki disease and infection with Influenza B or with other viruses such as coronavirus. Therefore, this association should be considered in pediatric patients, adolescents included, with prolonged febrile conditions.

## Abstract (pt)

Apresentação de um caso de infecção por Influenza B e doença de Kawasaki em adolescente ocorrido durante a pandemia da COVID-19. Adolescente asmática evoluiu com febre e síndrome gripal por 7 dias e deu entrada com quadro de insuficiência respiratória aguda, necessitando de intubação orotraqueal. Evoluiu também com instabilidade hemodinâmica respondedora ao uso de droga vasoativa. Foram introduzidas antibioticoterapia e medidas de suporte. Apresentou melhora hemodinâmica e respiratória progressiva, porém mantinha febre e alteração de provas inflamatórias. Durante internação, evoluiu com conjuntivite não purulenta bilateral, descamação de mão e pés, língua em framboesa e linfonodomegalia cervical, recebendo diagnóstico de doença de Kawasaki. Recebeu gamaglobulina e, por conta de quadro clínico refratário, foi administrado também corticoide, evoluindo afebril 24 horas após. Não apresentou alterações coronarianas. O único agente isolado foi Influenza B, mesmo realizando painel viral e investigação para COVID-19 com reação em cadeia da polimerase e sorologia. Durante internação, apresentou tromboembolismo pulmonar, e, em investigação de coagulopatias, foi diagnosticada com mutação em heterozigose de fator V de Leiden. Há uma potencial associação entre doença de Kawasaki e infecção por Influenza B ou outros vírus, como o coronavírus e, por isso, esses diagnósticos devem ser investigados nos pacientes pediátricos, incluindo adolescentes, com quadros febris prolongados.

## INTRODUCTION

The Kawasaki disease (KD) is a clinically diagnosed systemic vasculitis. Its diagnosis is defined by fever for five or more days, associated with four of five clinical criteria as follows: non-purulent conjunctivitis; strawberry tongue, oropharynx erythema or fissures and labial erythema; hands and feet erythema and/or edema with periungual peeling; morbilliform or polymorphic rash and cervical lymphadenopathy.^(1)^

Kawasaki disease’s etiology is unknown, but the main theories point to an association between infectious triggers in genetically susceptible children, as proposed by Chang et al. in a prospective case-control trial.^(2)^ Examples of infectious triggers are Influenza, adenovirus, respiratory syncytial virus, Epstein-Barr virus, and severe acute respiratory syndrome coronavirus 2 (SARS-CoV-2). In addition, the KD susceptibility and severity are influenced by variants in several different genes and signaling pathways. This polymorphism is variable across populations, and important differences in allele frequency are predicted, explaining the increased incidence of this disease in Asian populations.^(1)^

There are reports of KD in patients with Influenza infection, however, the association between Influenza B and KD is rare in the literature.

This case report complies with the Declaration of Helsinki and the Nuremberg Code precepts, and with the Brazilian National Health Council Standards for Research Involving Human Beings (resolution 466/12) and was approved by the Institution’s Research Ethics Committee. An Informed Consent Form was signed by the patient’s legal guardians.

## CASE REPORT

M.C.L.M., a 16-year-old female, presented with productive cough for seven days, followed by fever and progressive respiratory distress for four days. The patient had a history of asthma, crisis-free for one year. There were no other comorbidities, allergies, surgeries, or previous hospitalizations. Parents were healthy, and no suspect contacts were reported.

**Figure 1:**
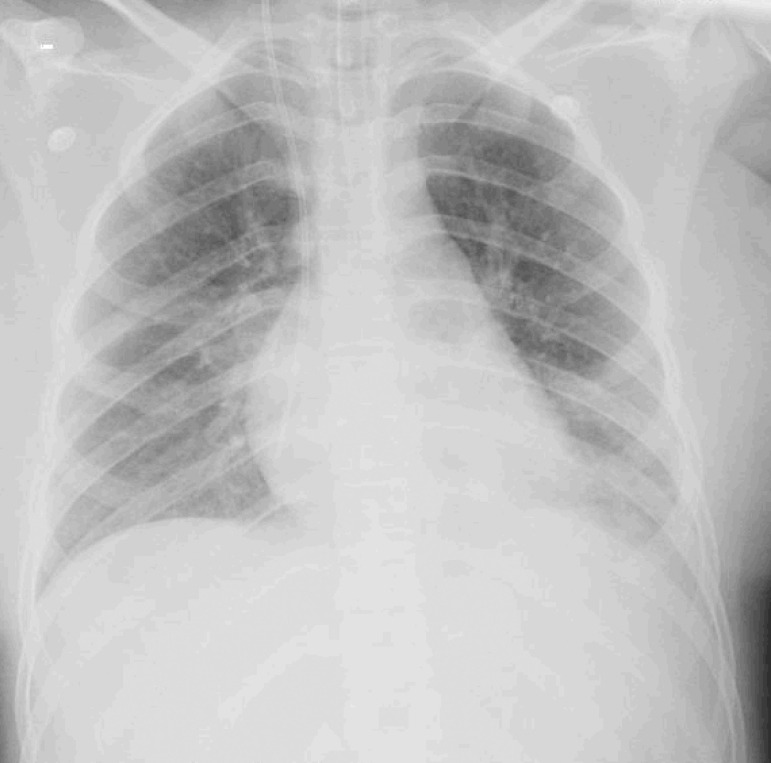
Admission chest radiography of a patient with Influenza B infection and subsequent diagnosis of Kawasaki disease.

She was seen at the beginning of symptoms and treated with azithromycin; on Day 7, she returned to the Emergency Department due to significantly worsened dyspnea and desaturation. She was treated for bronchospasm and was provided noninvasive ventilation, with poor response, and the patient was intubated and mechanically ventilated. Under sepsis protocol, she was given ceftriaxone (2g endovenous - EV - 12/12 hours) and oseltamivir (75mg orally 12/12 hours). Respiratory viruses panel plus C-reactive protein (CRP) for coronavirus disease 2019 (COVID-19), cultures, laboratory tests, and image tests were performed (Figure 1). On the same day, she was referred to the pediatric intensive care unit.

**Figure 2:**
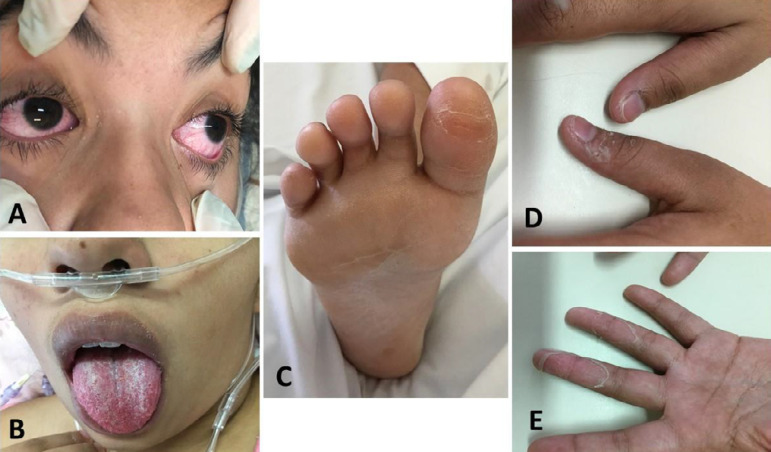
Conjunctivitis (A), oral changes (B), plantar (C), and palmar laminar desquamation (D and E), in a patient with Influenza B infection and Kawasaki disease.

The next day, tests resulted positive for Influenza B and negative for all other agents. She underwent ventilatory weaning and was extubated after 2 days. Norepinephrine was given for hemodynamic instability. On the third day of ceftriaxone, the patient persisted febrile and oxacillin (2g EV 4/4 hours) was added. On the eight day in the hospital, her general condition was improved, but she persisted febrile for 11 days, and laminar peeling in hands, feet, and the inguinal region was observed, in addition to a strawberry tongue (Figure 2). She had bilateral conjunctivitis (Figure 2A) since admission in addition to an increased left cervical lymph node (5cm), therefore completing the criteria for KD diagnosis. She was given acetylsalicylic acid (ASA) 200mg o.a.d. and one dose of immunoglobulin 80g EV, according to the protocol. Baseline and control echocardiograms showed no coronary changes (first echocardiogram: right coronary artery: 2.9mm and z-score -0.57, left coronary artery: 3.3mm and z-score -0.49, anterior descending artery: 3.2mm and z-score +1.0). Laboratory tests are shown in table 1.

**Table 1:** Laboratory tests

	Values	Laboratory reference
Hemoglobin (g/dL)	13.4	12 - 15.5
White blood cells count (µL)	26,180	3,500 - 10,500
Lymphocytes (µL)	785	900 - 2,900
CRP (mg/dL)	260	≤ 5
ESR (mm)	108	3 - 13
Procalcitonin (ng/mL)	0.56	< 0.5
D-dimer (ng/mL)	3,243	≤ 500
Interleukin 6 (pg/mL)	1,207	1.5 - 7
Troponin (pg/mL)	12	≤ 5
Ferritin (ng/mL)	350	3.88 - 114

CRP - C-reactive protein; ESR - erythrocyte sedimentation rate.

**Figure 3:**
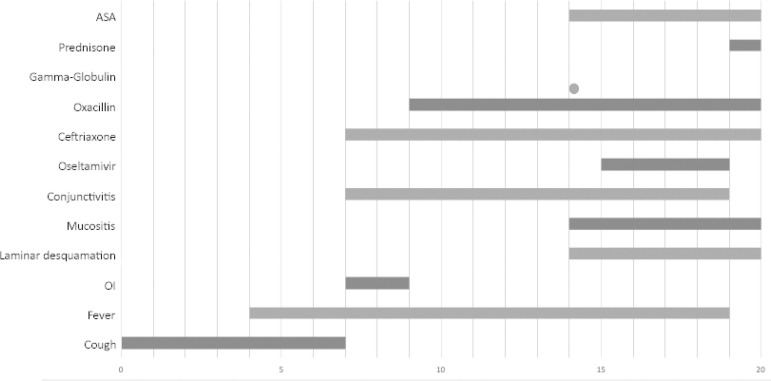
Symptoms and treatment of a patient with Influenza B infection and Kawasaki disease. ASA - acetylsalicylic acid; OI - orotracheal intubation.

Due to persistent tachypnea and worsened chest x-ray images, in addition to desaturation, a computed tomography angiography was performed, showing pulmonary thromboembolism, mild bilateral pleural effusion, and pulmonary infiltrates. Enoxaparin 40mg subcutaneous 12/12 hours was prescribed. Tests for systemic lupus erythematosus were negative, as well as serologies for cytomegalovirus disease, toxoplasmosis, and Epstein-Barr virus. On the 18th day of fever, prednisolone (60mg orally once a day) was prescribed. She was afebrile for the next 24 hours. Due to pulmonary thromboembolism, coagulopathies were investigated, and heterozygous factor V Leiden mutation was diagnosed. She was discharged to home with ASA (200mg orally o.a.d.) and warfarin (10mg orally o.a.d.), and referred to outpatient follow-up. During the follow-up, a COVID-19 serology tested negative more than 1 month after the symptoms started (Figure 3).

## DISCUSSION

During the global coronavirus pandemic, new concerns related to severe Kawasaki-like disease have arisen after complications related to multisystem inflammatory syndromes and KD outbreaks, and some cases were described of COVID-19 in association with this disease.^(3,4)^ This led the World Health Organization (WHO) to create a preliminary definition for multisystem inflammatory disorder secondary to COVID-19, later confirmed by the *Sociedade Brasileira de Pediatria* (SBP).^(5-6)^ The reported case was diagnosed before this new definition and is consistent with this worldwide trend of KD cases increase.

The etiology of KD is not fully explained, but many authors hypothesize an association with viral infections. Among the suspected agents, enteroviruses, adenoviruses, rhinoviruses, metapneumoviruses, and Influenza B were described.^(2)^ Regarding the influenza B virus, in 1993 Jackson et al.^(7)^ described a case of KD and Influenza B. Subsequently new reports correlated the Influenza virus with this disease, but reports involving the subtype B are rare.^(7,8)^ This case report raises the hypothesis of an association between KD and Influenza B virus infection.

Some authors argue that KD is caused by an endemic pathogen that, in a patient with a genetic predisposition, usually causes a mild and self-limiting disease, indistinguishable from other childhood viral diseases.^(8)^ Large prevalence studies have been conducted over several years and have shown that KD appears to follow seasonal epidemiological patterns. In southeastern Brazil, the prevalence of Influenza B infections starts to increase in March, and peaks during the winter.^(9)^ The reported patient was diagnosed in April, in agreement with this virus seasonality.

Kawasaki disease usually is found in children below 5 years old and the diagnosis in older children is often delayed.^(10)^ Despite this, in rare cases, it can be diagnosed in older children or even in adults. Therefore, another particular aspect of this report is related to the patient’s age - 16 years - outside the usual disease prevalence range. Older children diagnosed with KD have a more marked inflammatory response, are more likely to need more than one dose of immunoglobulin, have a higher prevalence of coronary abnormalities, and some of these patients, progress with myocarditis or hypotensive shock.^(10)^ The reported patient was diagnosed after 12 days with fever, close to the average 13.5 days reported by Jindal et al.^(10)^ Although this patient had no coronary changes, she had a vasoactive drugs-responsive hypotensive shock, persistent fever for 6 days after administration of immunoglobulin, having received adjuvant corticosteroid therapy from the fifth day on. These severe features are more prevalent in older children.

It is recommended that coronary arteries should undergo image diagnosis with quantitative assessment of the luminal dimensions using normalized z-scores adjusted to the body surface. According to the protocol, the echocardiography was reassessed after one and two weeks after treatment and continued during the follow-up. Despite no coronary changes were found, it is important to acknowledge the echocardiography limitations for KD patients’ assessment and follow-up. Although echocardiographic detection of thrombi and coronary artery stenosis have been reported, the echocardiography sensitivity and specificity for identification of these abnormalities are unclear. In addition, the visualization of coronary arteries becomes progressively difficult as the body size increases.^(1)^

It is important to highlight that this diagnosis occurred during the coronavirus pandemic and that the literature mentions possible associations between SARS-CoV-2 infection and multisystemic inflammatory syndrome, with a clinical presentation similar to KD. The portrayed patient had both CRP and serology negative for the SARS-CoV-2, which may be related to the time of collection or reflect a false-negative result.^(5)^

## CONCLUSION

This report supports the hypothesis of an association between Kawasaki disease and Influenza B infection and agrees with a worldwide increase of cases involving multisystemic inflammatory syndrome during the COVID-19 pandemic. These diagnostic possibilities should be kept in mind and investigated in pediatric patients with prolonged febrile conditions. In addition, this case highlights the importance of considering this diagnosis in adolescents, whose diagnosis is often delayed.
